# Possible Prognostic and Therapeutic Significance of c-Kit Expression, Mast Cell Count and Microvessel Density in Renal Cell Carcinoma

**DOI:** 10.3390/ijms150713060

**Published:** 2014-07-23

**Authors:** Ilaria Marech, Cosmo Damiano Gadaleta, Girolamo Ranieri

**Affiliations:** Diagnostic and Interventional Radiology Unit with Integrated Section of Translational Medical Oncology, National Cancer Research Centre Istituto Tumori “Giovanni Paolo II”, Via Orazio Flacco n° 65, 70100 Bari, Italy; E-Mails: i.marech@oncologico.bari.it (I.M.); c.gadaleta@oncologico.bari.it (C.D.G.)

**Keywords:** renal cell carcinoma, c-Kit receptor, stem cell factor, mast cells, microvessel density, molecular targeting agents

## Abstract

Renal cell carcinoma (RCC) is the most frequent renal tumor and its incidence is increasing worldwide. Tumor angiogenesis is known to play a crucial role in the etiopathogenesis of RCC and over the last few years an even deeper knowledge of its contribution in metastatic RCC development has led to the development of numerous molecular targeting agents (such as sunitinib, sorafenib, pazopanib, axitinib, tivozanib, and dovitinib). The above agents are principally directed against vascular endothelial growth factor receptor (VEGFR) members and also against c-Kit receptor (c-KitR). The role of c-kitR inhibition on clear cell RCC (ccRCC), the main RCC subtype, is less well established. Whether c-kitR activation through its ligand, stem cell factor (SCF) contributes significantly to the effects of tyrosine kinase inhibitors (TKIs) treatment remains to be established. It is important to underscore that the c-KitR is expressed on mast cells (MCs) and cancer cells. After an examination of the c-KitR/SCF pathway, we review here the principal studies that have evaluated c-Kit expression in RCC. Moreover, we summarize some investigations that have observed the distribution of MCs in primary renal cancer and in adjacent normal tissue with appropriate histological immunohistochemical techniques. We also focus on few studies that have evaluated the correlation between RCC proliferation, MC count and microvessel density (MVD), as hallmarks of tumor angiogenesis. Thus, the aim of this review of the literature is to clarify if c-KitR expression, MC count and MVD could have prognostic significance and the possible predictive therapeutic implications in RCC.

## 1. Introduction

Renal cell carcinoma (RCC) is the most frequent renal tumor and accounts for 3% of human cancers occurring in the world [1]. It is the cause of 95,000 deaths each year and represents the most lethal cancer within the urological neoplasms [2]. Its peak incidence is at around the sixth to seventh decade of life [1,2]. RCC is a neoplasm of epithelial nature, since it originates from the transformation of cells that constitute the epithelium of the proximal convoluted tubule [3]. Its principal histological types are: the clear cell RCC (75%–80%), papillary or chromophile RCC (10%–15%) and chromophobe RCC (4%–6%) [3].

**Figure 1:**
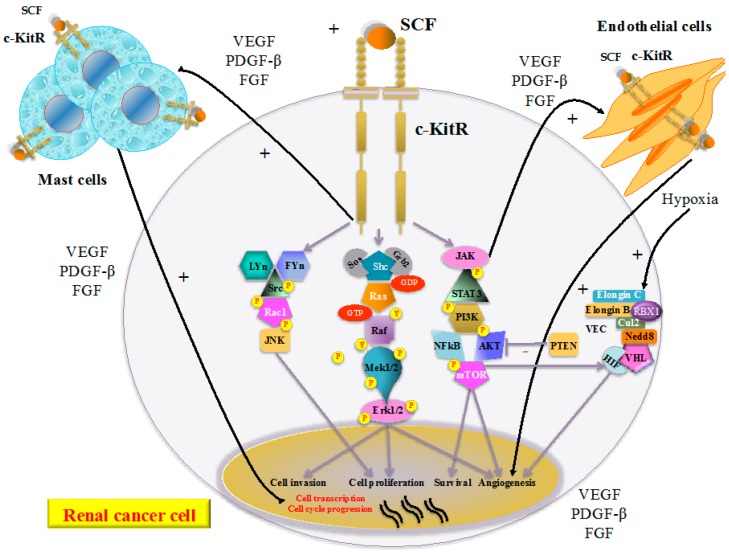
In renal cancer cells, stem cell factor (SCF) binding to the c-Kit receptor (c-KitR) induces several signal transduction pathways that lead to its proliferation, invasion, survival, and angiogenesis. Moreover, the increased activation of the c-KitR pathway leads to the activation of MC, which secretes pro-angiogenic cytokines (VEGF, PDGF-β, FGF) and induces cross-talk between MC, renal cancer cells and endothelial cells leading to the consequential strengthening of pro-angiogenic signaling,. In addition, hypoxia induces HIF to stimulate the transcription of genes that lead to the secretion of pro-angiogenic factors. Abbreviations: c-KitR, c-Kit receptor; SCF, stem cell factor, VEGF, vascular endothelial growth factor; PDGF-β, platelet derived growth factor-β; FGF, Fibroblast Growth Factor; *LYn*, *Lck/Yes*-related protein kinase; *FYn*, *FYn* oncogene related to SRC, FGR, YES; *Src*, V-*Src* sarcoma (Schmidt-Ruppin A-2) viral oncogene homolog; Rac1, Ras-related C3 botulinum toxin substrate 1; *JNK*, *c*-*Jun* *N*-terminal kinase *JNK*; Sos, son of sevenless; Grb2, growth factor receptor-bound protein 2; Shc, SHC-adaptor protein; Ras, rat sarcoma protein; *Raf*, *RAF* proto-oncogene serine/threonine-protein kinase; Mek1/2, MAP kinase-ERK kinase; Erk1/2, Elk-related tyrosine kinase; JAK, Janus kinase;STAT3, signal transducer and activator of transcription 3; PI3K, phosphatidyl inositol 3-kinase; NF-κB, nuclear factor kappa B; AKT, protein kinase B; mTOR, mammalian target of rapamycin; PTEN, phosphatase and TEnsiN homolog; VHL,von Hippel-Lindau protein; Cul2, cullin2; Nedd8, neural precursor cell expressed developmentally down-regulated 8; RBX1, ring-box 1; VEC, E3 ubiquitin ligase complex; HIF, hypoxia-inducible factor.

With reference to RCC etiopathogenesis, historically, among the most frequent chromosomal alterations involved in its onset are those that lead to the functional inactivation (due to the loss of both alleles) of the von Hippel-Lindau protein (pVHL), the product of the *VHL* tumor suppressor gene that is located on chromosomal region 3 p25–26 [4]. The functional form of the pVHL in association with elongin C, elongin B, cullin2 (Cul2), neural precursor cell expressed developmentally down-regulated 8 (Nedd8) and ring-box 1 (RBX1) forms a multi-protein complex called E3 ubiquitin ligase (or VEC) able in turn to bind the hydroxylated form of the subunit α of the transcription hypoxia-inducible factor (HIF) [4,5,6,7]. In normoxic conditions the formation of this complex leads to the degradation of HIF, while in case of hypoxia the stabilized form (non-hydroxylated) HIF is able to induce the transcription of genes that leads to the secretion of pro-angiogenic factors (such as vascular endothelial growth factor (VEGF) and platelet derived growth factor-β (PDGF-β)), Glucose transporter 1 (GLUT-1) and erythropoietin [4,5,6,7,8] (Figure 1). Over the last few years the ever-deeper knowledge on the molecular biology of metastatic RCC has led to the development of numerous molecular targeting agents (such as sunitinib, sorafenib, pazopanib, axitinib, tivozanib, and dovitinib) [9]. The above agents are principally directed against vascular endothelial growth factor receptor (VEGFR) members and also against the c-Kit receptor (c-KitR) [9]. The role of c-kitR inhibition on clear cell RCC (ccRCC), the main RCC subtype, is less well established [9]. Whether c-kitR activation through its ligand, stem cell factor (SCF) contributes significantly to the effects of tyrosine kinase inhibitors (TKIs) treatment remains to be established [9]. c-KitR is expressed on mast cells (MCs), endothelial and cancer cells. The c-KitR activation by means of its ligand, the stem cell factor (SCF), induces several signal transduction pathways, mitogen-activated protein kinase (MAPK) and phosphatidyl inositol 3-kinase (PI3K)/protein kinase B (AKT) (Figure 1) [9,10]. The increased activation of the c-KitR pathway leads in turn to MC activation, which secretes pro-angiogenic cytokines (such as VEGF, PDGF-β, and fibroblast growth factor (FGF)). The c-KitR activation in RCCs induces cross-talk between the cancer cells, MCs and endothelial cells, leading to the consequential strengthening of pro-angiogenic signaling.

It is thus well known that tumor angiogenesis plays a pivotal role in the etiopathogenesis of RCC [4,5,6,7,8,9,10]. Considering the pivotal role of c-KitR and the MC involvement in RCC progression, after a detailed examination of the c-KitR/SCF axis, the principal studies that have evaluated both c-KitR expression in RCC and the distribution of MCs in primary renal cancer and in adjacent normal tissue with appropriate histological techniques are analysed in the following paragraphs. In addition, we also focus on few studies that have assessed the correlation between RCC proliferation, MC count and microvessel density (MVD), as hallmarks of tumor angiogenesis, with the aim to clarify whether these variables could have prognostic significance and possible therapeutic implications in RCC.

## 2. c-Kit Receptor/Stem Cell Factor Pathway

The c-KitR is encoded by the c-Kit proto-oncogene localized on chromosome 4q and belongs to class III of the tyrosine kinase receptor (TKR) family [11,12]. The c-KitR and SCF (its ligand over-expressed in various inflammatory diseases) [13] regulate several physiological processes, including erythropoiesis, lymphopoiesis, megakaryopoiesis, gametogenesis, melanogenesis, and MC/eosinophil activations [12,14]. The c-KitR structure includes an extracellular region (consisting of five immunoglobulin-like domains), a trans-membrane (or JM) region, and an intracellular TK domain separated in two subdomains by an insert region (Figure 1) [15]. The interaction between c-KitR and SCF occurs through receptor phosphorylation and the formation of various homo/heterodimers with the activation of specific intracellular signaling pathways, including the janus kinase (JAK) and signal transducer and activator of transcription (STAT) pathway, the PI3K, AKT and mammalian target of rapamycin (mTOR) pathway, the MAPK pathway and V-*src* sarcoma (Schmidt-Ruppin A-2) viral oncogene homolog (Src) pathway [9]. Increased activation of the c-KitR pathways leads to the release of pro-angiogenic cytokines (VEGF, PDGF-β, FGF) from renal cancer cells, MCs and endothelial cell, inducing the strengthening of pro-angiogenic signaling, and in turn cancer cell survival, invasion and proliferation [9,15,16].

Two forms of c-KitR have been found: wild-type (145 kDa) and mutant-type (125 kDa) [17]. The deregulation and over-expression of the complex c-KitR signaling network, induced mainly by mutant-type form of c-KitR, have been discovered to be associated with cancer transformation in a variety of human malignancies [18,19,20,21,22,23,24]. C-KitR mutations can be present localized in the fifth extracellular domain (exon 8 and exon 9, e.g., Ala502–Tyr503 duplication specific in GIST), in the JM region (exon 11, e.g., V559D, a deletion of nine amino acids in the JM domain, called D27 mutant), and in the kinase domain (exon 17) [25]. Imatinib mesylate (Gleevec^®^, Basilea, Swiss), a selective c-KitR tyrosine kinase inhibitor (TKI), at first approved for the treatment of chronic myelogenous leukaemia (CML) and unresectable and/or malignant gastro-intestinal stromal tumor (GIST) [26], is now under investigation for the treatment of non-small cell lung cancer, ovarian cancer, Ewing’s sarcoma and melanoma [18,19,24,27,28]. However, some c-KitR mutations, especially those in exon 9 and exon 17 (the activating codon 816 Asp→Val mutation (D816V) frequently found in mastocytosis) are associated with resistance to imatinib mesylate therapy [25,29]. In fact, currently, other c-KitR TKIs, such as masitinib mesylate (AB1010) that inhibits mainly the wild-type and mutated c-KitR forms, are under evaluation in several clinical trials [30].

In order to determine whether selective c-KitR TKIs could be feasible in RCC, several studies have been conducted to examine c-Kit/c-KitR expression in different RCC histological subtypes [31,32,33,34,35,36,37,38,39].

## 3. The Significance of c-Kit Expression in Renal Cancer Patients

Considering the biologic background of c-KitR in RCC tumorigenesis, the overexpression of c-KitR may be involved in tumor development and progression; therefore c-KitR could represent an intriguing therapeutic target in these patients.

Over the years, several authors had examined c-Kit/c-KitR expression in different histological subtypes of RCC [31,32,33,34,35,36,37,38,39] all concluding that the chromophobe variety (and also the oncocytoma among benign renal tumors) had frequent c-KitR expression of strong [9,31] or moderate [32] intensity in immunohistochemistry (IHC) compared to other malignant histological subtypes, such as papillary and clear cell [31,32,33,34,35,36,37,38,39] (Table 1). In agreement, they attributed to increased c-Kit/c-KitR expression in chromophobe RCC variety a possible therapeutic target besides a diagnostic significance, as tumor biomarker [31,32,33,34,35,36,37,38,39]. Yamazaki *et al.* emphasized the observed strong c-KitR over-expression in chromophobe variety of RCC with the purpose to clarify the nature of its tumorigenesis, considering that no gene mutations have been noted related to the development of chromophobe variety compared to the well known *VHL* and mesenchymal-epidermal transition (*MET*) genetic mutations involved in the tumorigenesis of clear cell and papillary RCCs, respectively [31]. Huo *et al.* demonstrated c-Kit over-expression in both chromophobe RCC (and oncocytoma) at the mRNA level by cDNA microarray analysis also, confirming in angiomyolipoma, papillary, and clear cell RCC a low c-Kit expression [36]. Considering the frequency of c-Kit/c-KitR expression in other histological subtypes of RCC (clear cell and papillary) all authors were in agreement to discover its slight [31], low (between 2% and 3% in clear cell and 5%–7% in papillary) [35,36,38] or absent [32,33,37,39] expression. Curiously, Sengupta *et al.* showed the presence of c-KitR expression only in 4% of RCC with sarcomatoid differentiation [38]. Wang *et al.* suggested that the presence of c-KitR expression (found in 100% of chromophobe cases) could be the hallmark of chromophobe variety being absent in all granular cell variants of clear cell RCC [34].

With special reference to c-KitR expression staining pattern, several authors demonstrated chromophobe varieties that can be simultaneously membranous and cytoplasmic [9,32,34,36,37], mainly membranous [32,37] or only membranous [31,38,39]. Interestingly, Horstmann *et al.* found a significantly higher membranous c-KitR expression staining pattern in clear cell and papillary varieties compared to both chromophobe variety and normal renal tissue (*p* < 0.05) [9]. Curiously, the authors assessed also SCF expression which correlated with cytoplasmic c-KitR in all histological types (*p* < 0.01); moreover its expression was higher in oncocytoma rather than in clear cell and chomophobe varieties (*p* < 0.05) [9]. The authors hypothesize that concomitant expression of SCF and c-KitR, which seems to undergo a shift from the cytoplasm to the cell membrane, suggests paracrine and autocrine mechanisms in c-KitR activation with distinct regulatory mechanisms in the different tumor varieties [9]. Regarding c-KitR expression pattern as prognostic factor, Horstmann *et al.* showed a significant correlation between advanced stage (pT3) and low cytoplasmic c-KitR expression (*p* < 0.05) [9]. Kruger *et al.* also found a significant correlation between advanced stage (pT ≥ 2) and low cytoplasmic c-KitR expression (*p* = 0.036) [37]. With reference to tumor grade and histological type, the proportion of cases with cytoplasmic c-KitR staining was higher in G2 tumors (78%) and in tumors of the classic variant (82%) compared to G3 tumors (50%) and to tumors of the eosinophilic variant (67%), even if the difference was not statistically significant (*p* = 0.376 and *p* = 0.331, respectively) [37].

**Table 1:** Principal studies evaluating c-Kit/c-KitR expression in renal cancer patients.

Author, Reference, Year	Histological Types (%)		Stage	Patients (*n*)	Methods of c-Kit/c-KitR Evaluation		Type of c-KitR Pattern		Percentage of c-Kit/c-KitR Expression		Intensity of c-Kit/c-KitR Expression
Yamazaki [31] 2003	clear cell: 67% chromophobe: 20% papillary: 13%		n.d.	15	immunohistochemistry primary MoAb anti-CD 117		membranous		n.d.		chromophobe: strong clear cell: slight papillary: slight
Zigeuner [32] 2004	clear cell: 76% chromophobe: 13% papillary: 11%		I–IV	180	immunohistochemistry primary PoAb anti-CD 117		mainly membranous		n.d.		chromophobe: moderate clear cell: none papillary: none
Petit [33] 2004	clear cell: 33% chromophobe: 29% oncocytoma: 15% papillary: 11%		n.d.	87	immunohistochemistry primary PoAb anti-CD 117		cytoplasmic or membranous or nuclear		chromophobe: 88% oncocytoma: 71% clear cell: 0% papillary: 0%		chromophobe: strong oncocytoma: moderate
Wang [34] 2005	oncocytoma: 52% chromophobe: 48%		n.d.	23	immunohistochemistry primary PoAb anti-CD 117		cytoplasmic or membranous		chromophobe: 100% oncocytoma: 100%		n.d.
Li [35] 2005	clear cell: 33% papillary: 33% oncocytoma: 18% chromophobe: 16%		I–IV	45	RT-PCR c-Kit gene expression		electrophoresis band as DNA marker		chromophobe: 86% oncocytoma: 88% papillary: 7% clear cell: 0%		n.d.
Huo [36] 2005	clear cell: 23% oncocytoma: 24% chromophobe: 23% angiomyolipoma: 12% papillary: 9%		n.d.	171	immunohistochemistry primary MoAb anti-CD 117		cytoplasmic or membranous or both		chromophobe: 95% oncocytoma: 88% angiomyolipoma: 17% papillary: 5% clear cell: 3%		n.d.
Kruger [37] 2005	chromophobe: 39% clear cell: 27% oncocytoma: 18% papillary: 7%		I–III	74	immunohistochemistry primary PoAb anti-CD 117		membranous or both		chromophobe: 100%–77% oncocytoma: 100%–77% clear cell: 0% papillary: 0%		n.d.
Sengupta [39] 2006	clear cell: 90.2% chromophobe: 3.6% n.s.: 3.1% papillary: 1.6%		n.d.	194	immunohistochemistry primary PoAb anti-CD 117		membranous		n.s.: 33.3% chromophobe: 14.3% clear cell: 2.3% papillary: 0%		n.d.
Terada [39] 2012	clear cell: 70% chromophobe: 15% papillary: 15%		n.d.	61	immunohistochemistry primary MoAb anti-CD 117		membranous		chromophobe: 100% clear cell: 0% papillary: 0%		n.d.
Horstmann [9] 2012	clear cell: 36% oncocytoma: 24% papillary: 23% chromophobe: 17%		I–IV	111	immunohistochemistry primary PoAb anti-CD 117		cytoplasmic or both		n.d.		chromophobe: strong oncocytoma: strong papillary: strong/moderate clear cell: slight/moderate

MoAb, monoclonal antibody; PoAb, polyclonal antibody; n.d., not determined; RT-PCR, reverse transcriptase-polymerase chain reaction.

Finally, considering the mutational status of c-Kit, pilot studies have addressed this by detailed analyses; interestingly, as all authors showed, in all cases examined not a single mutation was revealed [31,37,38,39]. Yamazaki *et al.* demonstrated no mutation in the region through sequencing analysis of the c-Kit gene in the three chromophobe RCCs [31]. Kruger *et al.* using total DNA from 38 tumors (47% chromophobe, 32% oncocytomas, 11% clear cells) in a c-Kit mutation analysis showed no mutations, in particular the D816V mutation [37]. Sengupta *et al.* identified no mutations in all immunopositive (only seven grade 4 chromophobe variety) cases by polymerase chain reaction amplification of c-Kit exons 9, 11, 13 and 17 [38]. Terada *et al.* also showed no mutations of c-Kit exons 9, 11, 13 and 17 in all 30 cases analyzed using PCR-direct sequencing [39].

## 4. The Significance of Mast Cells and Angiogenesis in Renal Cancer Patients

MCs originate from CD34+ hematopoietic stem cells and require SCF for their activation [40]. MCs are located throughout the body and mainly near blood vessels [40]. Classically, they are divided into three subgroups according to the protease expression in their granules: the first type of MC contains only tryptase, the second only chymase, and the third tryptase, chymase and other proteases [41]. The role of MC has long been well defined at first in hypersensitivity reactions, and then in both innate and adaptive immunity [42,43]. This has allowed us to redefine their crucial interplay on the regulatory function between inflammatory and tumor cells [44,45,46,47] by means of the release of various granule-associated mediators (tryptase, chymase, tumour necrosis factor (TNF), VEGF, FGF, PDGF-β, epidermal growth factor (EGF)), lipid-derived mediators (leukotrienes, prostaglandins), cytokines (transforming growth factor-β (TGF-β), interleukins), and chemokines [48,49,50,51]. The pivotal involvement of MC in tumorigenesis has emerged from observation of a strong correlation between an increase of MC count and an increase of microvascular density (MVD) in many human malignancies [43,44,46,48,52,53,54,55,56,57,58,59,60,61,62,63,64,65].

In normal human kidney, there are few resident MCs to be observed in the interstitium of the renal cortex around blood vessels or between renal tubules [66,67]. Historically, already both Staemmler and Lascano found an increase of MC count in the interstitium of some cases of hypernephroma [68,69]. In 1998, Beil *et al.* characterized the phenotype and functional properties of MCs obtained from renal tumors observing that they contained mainly tryptase, c-KitR, surface IgE, CD43, CD44, CD54, and CD63 [70].

In RCC the relationship between MC and angiogenesis has not yet been well defined because of few studies have been conducted in order to establish type, number and location of MCs in renal tumor microenvironment and the correlation between MC count and tumor angiogenesis [71,72]. However, the majority of studies showed an increase of MC count in renal tissue compared to normal renal tissue [71,72], thus hypothesizing a close interplay between MC and renal cancer cell in tumorigenesis. In agreement with this evidence, MC count could represent a prognostic factor.

Concerning a possible prognostic significance of MVD in RCC, the majority of studies found that high MVD is related to poor prognosis [73,74], whereas other studies found no prognostic value of MVD [75,76].

Tuna *et al.* showed that MC count was correlated with MVD (*p* = 0.034) but not with prognosis (tumor stage, grade, size), and patient survival. A tendency has been observed (even if not statistically significant) for increased MC count with increasing tumor stage. Higher MC count was observed in 14.8% of early-stage tumors than in 33.4% of advanced-stage tumors. MC count was significantly higher in clear as compared to non-clear RCC (*p* = 0.034) [71]. Moreover, MC count was increased in RCC and peritumoral tissue with inflammation compared to that normal renal tissue (*p* < 0.001) [71]. Therefore, this evidence confirms that MVD does not appear to be a useful prognostic factor in RCCs. On the one hand MVD seems not to be a prognostic factor in RCC, but on the other, MC count may be related to tumor angiogenesis and tumorigenesis in RCC [71].

Mohseni *et al.* also demonstrated that MCs were mainly observed in both tumor and peritumoral inflammatory renal tissue, whereas in non-tumoral renal tissue it has been found that MCs sprinkled the interstitium of the cortex or subcortical layer [72]. Moreover, MC count was significantly higher in RCC compared to that in normal renal tissue (*p* value not determined) [72]. The increase of MC count in renal cancer tissue compared to normal renal tissue could suggest an involvement of MC in stimulating angiogenesis-mediated renal cancer cell proliferation. However, it has also been shown that MC count did not correlate to MVD (*p* = 0.45) and prognosis (tumor stage, grade, size) [72]. The authors hypothesize that the weakness of this evidence is related to the selection of heterogeneous tumor groups in terms of tumor stage and grade and to MC identification methods (e.g., different primary antibodies employed and counting technique) [72].

Table 2 summarizes two studies that correlate MCs with MVD in renal cancer patients. Mclennan *et al.* showed no correlation between MVD and prognosis referring to clinical stage, pathologic stage, tumor grade, and cancer-specific survival (all *p* > 0.05). Interestingly, MVD was higher in the clear cell carcinoma compared to non-clear cell carcinoma (*p* = 0.007) [75].

Yoshino *et al.* observed a strong correlation between MVD and prognosis referring to disease-free survival mainly (*p* = 0.004) in patients with primary RCC. In particular, patients with 30 microvessels per 200× field had a higher survival than those more than 30 microvessels per 200× field (*p* = 0.007). Patients with metastatic disease had more hypervascular tumors than those who had non-metastatic disease (*p* = 0.0006) [73].

Nativ *et al.* observed a strong association between MVD and prognosis (as survival rate) (*p* = 0.00014). In fact, the 10-year survival rate for patients with low and high MVD was 91% and 46%, respectively (*p* = 0.041). However, there was an inverse correlation between MVD and some histopathological features including nucleus type (nuclear area, elipticity and roughness; respectively *p* = 0.006; *p* = 0.016; *p* = 0.039) and grading (*p* = 0.047) [74].

Kirkali *et al.* demonstrated no correlation between MVD and prognosis represented by mean nuclear volume, stage and survival (*p* > 0.05). Conversely, Fuhrman (*p* = 0.0011) and WHO grades (0.001 < *p* < 0.0002), and tumor stage (*p* = 0.0003) have all been confirmed as prognostic factors related significantly with patients’ survival [76].

Table 3 summarizes all studies that correlate MVD with prognosis in renal cancer patients.

**Table 2:** Studies correlating mast cell (MC) count with microvascular density (MVD) in renal cancer patients.

Author, Reference, Year	Histological Types	Stage	Patients (*n*)	Methods of MC Identification	Methods of MVD Identification	Correlation between MC Count & MVD	*p* Value
Tuna [71] 2006	clear cell: 66.2% chromophobe: 14.1% papillary: 11.3% sarcomatoid: 8.5%	I–IV	71	histochemistry toludine blue	immunohistochemistry primary MoAb anti-CD31	yes	*p* = 0.034
Mohseni [72] 2010	clear cell: 72.5% granular cell: 12.5% sarcomatoid: 7.5% chromophobe: 5% papillary: 1%	I–IV	40	histochemistry toludine blue	immunohistochemistry primary MoAb anti-CD34	no	*p* = 0.45

MoAb, monoclonal antibody.

**Table 3:** Studies correlating microvascular density (MVD) with prognosis in renal cancer patients.

Author, Reference, Year	Histological Types	Stage	Patients (*n*)	Methods of MVD Identification	Clinical Parameters	Correlation	*p* Value
Mclennan [75] 1995	clear cell: 75% granular cell: 11% papillary: 9% sarcomatoid: 3% chromophobe: 1%	I–IV	97	immunohistochemistry primary MoAb anti-factor VIII	DFS	no	*p* > 0.05
Yoshino [73] 1995	n.d.	I–IV	84	immunohistochemistry primary MoAb anti-factor VIII	DFS	yes	*p* < 0.004
Nativ [74] 1997	non papillary: 86.1% papillary: 13.9%	I–II	36	immunohistochemistry primary MoAb anti-factor VIII	OS	yes	*p* = 0.00014
Kirkali [76] 2001	clear cell: 60% chromophobe: 20% sarcomatoid: 13% chromophilic: 7%	I–IV	70	immunohistochemistry primary MoAb anti-CD31	OS, DFS	no	*p* > 0.05

MoAb, monoclonal antibody; DFS, disease free survival; OS, overall survival.

## 5. Conclusions

RCC represents the most lethal cancer within the urological neoplasms [2] accounting for 3% of human cancers occurring in the world [1].

Considering RCC etiopathogenesis, the most important reason for its onset is due to the inactivation of pVHL [4]. Normally, during normoxia pVHL leads to the degradation of HIF, while in case of hypoxia or its inactivation leads HIF to induce the secretion of pro-angiogenic factors (VEGF, PDGF-β, FGF) [4,5,6,7,8]. Increasing knowledge of the molecular biology of metastatic RCC has led to the development of several TKIs [9]. These TKIs are principally directed against VEGFR members and also against the c-Kit receptor (c-KitR). The role of c-kitR inhibition on clear cell RCC (ccRCC), the main RCC subtype, is less well established. Whether c-kitR activation through its ligand, stem cell factor (SCF) contributes significantly to the effects of tyrosine kinase inhibitors (TKIs) treatment remains to be established. It is important to underline that the c-KitR is expressed on MCs, endothelial cells, and cancer cells. The connection between c-KitR and SCF induces several signal transduction pathways in RCC that, in turn, promotes the activation of both MCs and endothelial cells, leading to the further strengthening of pro-angiogenic signaling.

With particular reference to the pivotal role of c-KitR in RCC progression, the selective c-KitR TKI imatinib mesylate (Gleevec^®^), currently approved for the treatment of CML and GIST [26], is now under investigation for the therapy of RCC in combination with everolimus (ClinicalTrials.gov Identifier: NCT00331409) or with bevacizumab and erlotinib (ClinicalTrials.gov Identifier: NCT00193258) for use also with other malignant tumors [18,19,20,21,22,23,24].

In order to determine whether c-KitR could be a tumor biomarker, all authors were in agreement to discover in chromophobe varieties frequent (from 77% to 100% of cases) c-KitR expression of strong [9,31] or moderate [32] intensity at IHC compared to other malignant histological subtypes (papillary and clear cell) [31,32,33,34,35,36,37,38,39]. Regarding c-KitR staining pattern in chromophobe variety (mainly and simultaneously membranous and cytoplasmic) [9,32,34,36,37], only Horstmann and Kruger *et al.* correlated c-KitR expression intensity or pattern to other variables (SCF expression, stage, histological subtypes, grading) hypothesizing that different c-KitR staining pattern depended on different c-KitR activation related to histological subtype [9,37]. Surprisingly, no authors demonstrated a biologic significance of the c-KitR staining pattern (as demonstrated in other tumors) [77] or a relationship between prognosis and c-KitR expression, MC count and MVD in RCC. Considering the mutational status of c-Kit, all authors showed in different histological subtypes assessed the absence of c-Kit mutation (in exons 9, 11, 13 and 17) [31,37,38,39]. Therefore, the absence of c-Kit mutations in the presence of its expression could confer to c-KitR an intriguing therapeutic significance as marker predictive of response to c-KitR TKIs, such as imatinib or masitinib. However, the only one phase II trial assessing imatinib alone in fourteen patients with metastatic RCC demonstrated poor efficacy of this treatment [78]. The reason could be due to only one tumor that demonstrated strong and diffuse expression compared to the rest, which were negative for c-KitR expression. Therefore, it is important to underline that initially it will be necessary conduct further clinical trials to evaluated c-KitR TKIs in selected patients (positive for c-KitR expression in IHC, harbouring the orphan chromophobe variety) rather than to evaluate the optimal sequential therapeutic strategy (e.g., prolonging the use of TKIs or using m-TOR inhibitors early after TKIs) in all patients [79].

Few and more controversial are the data relating to MCs and MVD. In half of the studies, independently assessing both parameters, the increased MC count and high MVD in RCC tissue could represent poor prognostic factors [71,73,74]. These controversial results could depend on the chosen heterogeneous tumor groups (in terms of tumor stage and grade, histological subtypes) and the methods of MC/MVD identification [72]. However, Tuna *et al.* found a significant correlation between increased MCs and clear cell variety and a not statistically significant tendency for high MC count with increased tumor stage [71]. Yoshino *et al*., [73], and Nativ *et al.* [74], showed a significant correlation between high MVD, low disease free survival (DFS), low overall survival (OS), metastatic disease and low grading [73,74]. According to Nativ *et al.* a possible explanation of the inverse relation between MVD and grading was that tumoral angiogenesis, which may facilitate metastatic spread, required more differentiated tumor cells, which can produce specific pro-angiogenic factors [74].

In conclusion, c-Kit (c-KitR/c-Kit) overexpression has been well demonstrated in chromophobe variety of RCC. Based on this overexpression, it is possible to hypothesize a role as tumor biomarker with diagnostic significance, although the level of evidence remains unclear. Considering the absence of c-Kit mutations in chromophobe variety, c-Kit (c-KitR/c-Kit) overexpression could represent a predictive factor of response to TKIs (imatinib or masitinib) worthy of further investigation. Moreover, MC count and MVD are surrogates of tumoral angiogenesis, but data to consider them as prognostic factors are not conclusive and need to be further investigated.

## References

[1] Ridge C.A., Pua B.B., Madoff D.C. (2014). Epidemiology and staging of renal cell carcinoma. Semin. Interv. Radiol..

[2] Ljungberg B., Campbell S.C., Cho H.Y., Jacqmin D., Lee J.E., Weikert S., Kiemeney L.A. (2011). The epidemiology of renal cell carcinoma. Eur. Urol..

[3] Srigley J.R., Delahunt B., Eble J.N., Egevad L., Epstein J.I., Grignon D., Hes O., Moch H., Montironi R., Tickoo S.K. (2013). The International Society of Urological Pathology (ISUP) vancouver classification of renal neoplasia. Am. J. Surg. Pathol..

[4] Vanharanta S., Shu W., Brenet F., Hakimi A.A., Heguy A., Viale A., Reuter V.E., Hsieh J.J., Scandura J.M., Massagué J. (2013). Epigenetic expansion of VHL–HIF signal output drives multiorgan metastasis in renal cancer. Nat. Med..

[5] Shen C., Kaelin W.G. (2013). The VHL–HIF axis in clear cell renal carcinoma. Semin. Cancer Biol..

[6] Zhang Q., Yang H. (2012). The roles of VHL-dependent ubiquitination in signaling and cancer. Front. Oncol..

[7] Medina Villaamil V., Aparicio Gallego G., Santamarina Caínzos I., Valladares-Ayerbes M., Antón Aparicio L.M. (2012). Searching for HIF1-α interacting proteins in renal cell carcinoma. Clin. Transl. Oncol..

[8] Billemont B., Méric J.B., Izzedine H., Taillade L., Sultan-Amar V., Rixe O. (2007). Angiogenesis and renal cell carcinoma. Bull. Cancer.

[9] Horstmann M., Hennenlotter J., Geiger L.M., Vogel U., Schmid H., Kuehs U., Stenzl A., Bedke J. (2012). Evaluation of the Kit/stem cell factor axis in renal tumours. Anticancer Res..

[10] Reith A.D., Ellis C., Lyman S.D., Anderson D.M., Williams D.E., Bernstein A., Pawson T. (1991). Signal transduction by normal isoforms and W mutant variants of the Kit receptor tyrosine kinase. EMBO J..

[11] Cools J., de Angelo D.J., Gotlib J., Stover E.H., Legare R.D., Cortes J., Kutok J., Clark J., Galinsky I., Griffin J.D. (2003). A tyrosine kinase created by fusion of the *PDGFRA* and *FIP1L1* genes as a therapeutic target of imatinib in idiopathic hypereosinophilic syndrome. N. Engl. J. Med..

[12] Ranieri G. (2012). Hot topic: Targeting tumor angiogenesis: An update. Curr. Med. Chem..

[13] Ribatti D., Ranieri G., Basile A., Azzariti A., Paradiso A., Vacca A. (2012). Tumor endothelial markers as a target in cancer. Expert Opin. Ther. Targets.

[14] Broudy V.C. (1997). Stem cell factor and hematopoiesis. Blood.

[15] Nick H.J., Kim H.G., Chang C.W., Harris K.W., Reddy V., Klug C.A. (2012). Distinct classes of c-Kit-activating mutations differ in their ability to promote RUNX1–ETO-associated acute myeloid leukemia. Blood.

[16] London C.A., Galli S.J., Yuuki T., Hu Z.Q., Helfand S.C., Geissler E.N. (1999). Spontaneous canine mast cell tumors express tandem duplications in the proto-oncogene c-Kit. Exp. Hematol..

[17] Broudy V.C., Lin N.L., Sabath D.F. (2001). The fifth immunoglobulin-like domain of the kit receptor is required for proteolytic cleavage from the cell surface. Cytokine.

[18] López-Martin A., Ballestín C., Garcia-Carbonero R., Castaño A., Lopez-Ríos F., López-Encuentra A., Sánchez-Cespedes M., Castellano D., Bartolomé A., Cortés-Funes H. (2007). Prognostic value of Kit expression in small cell lung cancer. Lung Cancer.

[19] Chau W.K., Ip C.K., Mak A.S., Lai H.C., Wong A.S. (2013). c-Kit mediates chemoresistance and tumor-initiating capacity of ovarian cancer cells through activation of Wnt/β-catenin-ATP-binding cassette G2 signaling. Oncogene.

[20] Wiesner C., Nabha S.M., dos Santos E.B., Yamamoto H., Meng H., Melchior S.W., Bittinger F., Thüroff J.W., Vessella R.L., Cher M.L. (2008). c-Kit and its ligand stem cell factor: Potential contribution to prostate cancer bone metastasis. Neoplasia.

[21] Cohen P.S., Chan J.P., Lipkunskaya M., Biedler J.L., Seeger R.C. (1994). Expression of stem cell factor and c-Kit in human neuroblastoma. The Children’s Cancer Group. Blood.

[22] Hassan S., Kinoshita Y., Kawanami C., Kishi K., Matsushima Y., Ohashi A., Funasaka Y., Okada A., Maekawa T., He-Yao W. (1998). Expression of proto-oncogene *c-Kit* and its ligand stem cell factor (SCF) in gastric carcinoma cell lines. Dig. Dis. Sci..

[23] Hines S.J., Organ C., Kornstein M.J., Krystal G.W. (1995). Co-expression of the c-kit and stem cell factor genes in breast carcinomas. Cell Growth Differ..

[24] Inoue M., Kyo S., Fujita M., Enomoto T., Kondoh G. (1994). Co-expression of the c-Kit receptor and the stem cell factor in gynecological tumors. Cancer Res..

[25] Orfao A., Garcia-Montero A.C., Sanchez L., Escribano L. (2007). Recent advances in the understanding of mastocytosis: The role of Kit mutations. Br. J. Haematol..

[26] Buchdunger E., Zimmermann J., Mett H., Meyer T., Müller M., Druker B.J., Lydon N.B. (1996). Inhibition of the Abl protein–tyrosine kinase *in vitro* and *in vivo* by a 2-phenylaminopyrimidine derivative. Cancer Res..

[27] Merchant M.S., Woo C.W., Mackall C.L., Thiele C.J. (2002). Potential use of imatinib in Ewing’s sarco-ma: Evidence for *in vitro* and *in vivo* activity. J. Natl. Cancer Inst..

[28] Hodi F.S., Corless C.L., Giobbie-Hurder A., Fletcher J.A., Zhu M., Marino-Enriquez A., Friedlander P., Gonzalez R., Weber J.S., Gajewski T.F. (2013). Imatinib for melanomas harboring mutationally activated or amplified Kit arising on mucosal, acral, and chronically sun-damaged skin. J. Clin. Oncol..

[29] Ma Y., Zeng S., Metcalfe D.D., Akin C., Dimitrijevic S., Butterfield J.H., McMahon G., Longley B.J. (2002). The c-Kit mutation causing human mastocytosis is resistant to STI571 and other Kit kinase inhibitors; kinases with enzymatic site mutations show different inhibitor sensitivity profiles than wild-type kinases and those with regulatory-type mutations. Blood.

[30] Marech I., Patruno R., Zizzo N., Gadaleta C., Introna M., Zito A.F., Gadaleta C.D., Ranieri G. (2014). Masitinib (AB1010), from canine tumor model to human clinical development: Where we are?. Crit. Rev. Oncol. Hematol..

[31] Yamazaki K., Sakamoto M., Ohta T., Kanai Y., Ohki M., Hirohashi S. (2003). Over-expression of Kit in chromophobe renal cell carcinoma. Oncogene.

[32] Zigeuner R., Ratschek M., Langner C. (2005). Kit (CD117) immunoreactivity is rare in renal cell and upper urinary tract transitional cell carcinomas. BJU Int..

[33] Petit A., Castillo M., Santos M., Mellado B., Alcover J.B., Mallofré C. (2004). Kit expression in chromophobe renal cell carcinoma: Comparative immunohistochemical analysis of Kit expression in different renal cell neoplasms. Am. J. Surg. Pathol..

[34] Wang H.Y., Mills S.E. (2005). Kit and RCC are useful in distinguishing chromophobe renal cell carcinoma from the granular variant of clear cell renal cell carcinoma. Am. J. Surg. Pathol..

[35] Li G., Gentil-Perret A., Lambert C., Genin C., Tostain J. (2005). *S100A1* and *Kit* gene expressions in common subtypes of renal tumours. Eur. J. Surg. Oncol..

[36] Huo L., Sugimura J., Tretiakova M.S., Patton K.T., Gupta R., Popov B., Laskin W.B., Yeldandi A., Teh B.T., Yang X.J. (2005). *c-Kit* expression in renal oncocytomas and chromophobe renal cell carcinomas. Hum. Pathol..

[37] Krüger S., Sotlar K., Kausch I., Horny H.P. (2005). Expression of Kit (CD117) in renal cell carcinoma and renal oncocytoma. Oncology.

[38] Sengupta S., Cheville J.C., Corless C.L., Lohse C.M., Heinrich M.C., Kwon E.D., Zincke H., Blute M.L., Leibovich B.C. (2006). Rare expression of Kit and absence of Kit mutations in high grade renal cell carcinoma. J. Urol..

[39] Terada T. (2012). Protein expression and gene mutation status of *Kit* and *PDGFRA* in renal cell carcinoma. Histol. Histopathol..

[40] Shea-Donohue T., Stiltz J., Zhao A., Notari L. (2010). Mast cells. Curr. Gastroenterol. Rep..

[41] Irani A.M., Schechter N.M., Craing S.S., de Blois G., Schwartz L.B. (1986). Two types of human mast cells that have distinct neutral protease composition. Proc. Natl. Acad. Sci. USA.

[42] Marshall J.S. (2004). Mast-cell responses to pathogens. Nat. Rev. Immunol..

[43] Mangia A., Malfettone A., Rossi R., Paradiso A., Ranieri G., Simone G., Resta L. (2011). Tissue remodelling in breast cancer: Human mast cell tryptase as an initiator of myofibroblast differentiation. Histopathology.

[44] Ranieri G., Ammendola M., Patruno R., Celano G., Zito F.A., Montemurro S., Rella A., di Lecce V., Gadaleta C.D., de Sarro G. (2009). Tryptase-positive mast cells correlate with angiogenesis in early breast cancer patients. Int. J. Oncol..

[45] Ranieri G., Labriola A., Achille G., Florio G., Zito A.F., Grammatica L., Paradiso A. (2002). Microvessel density, mast cell density and thymidine phosphorylase expression in oral squamous carcinoma. Int. J. Oncol..

[46] Ranieri G., Roccaro A.M., Vacca A., Ribatti D. (2006). Thymidine phosphorylase (platelet-derived endothelial cell growth factor) as a target for capecitabine: From biology to the bedside. Recent Pat. Anticancer Drug Discov..

[47] Passantino L., Patruno R., Valerio P., Penna A., Mazzone F., Zito A.F., Catalano V., Pellecchia A., Jirillo E., Ranieri G. (2005). Thymidine phosphorylase profiles in nonmalignant and malignant pancreatic tissue. Potential therapeutic role of capecitabine on tumoral and endothelial cells and tumor-infiltrating macrophages. Immunopharmacol. Immunotoxicol..

[48] Raica M., Cimpean A.M., Ceausu R., Ribatti D., Gaje P. (2013). Interplay between mast cells and lymphatic vessels in different molecular types of breast cancer. Anticancer Res..

[49] Ribatti D., Nico B., Finato N., Crivellato E. (2011). Tryptase-positive mast cells and CD8-positive T cells in human endometrial cancer. Pathol. Int..

[50] Nagata M., Shijubo N., Walls A.F., Ichimiya S., Abe S., Sato N. (2003). Chymase-positive mast cells in small sized adenocarcinoma of the lung. Virchows Arch..

[51] Horny H.P., Greschniok A., Jordan J.H., Menke D.M., Valent P. (2003). Chymase expressing bone marrow mast cells in mastocytosis and myelodysplastic syndromes: An immunohistochemical and morphometric study. J. Clin. Pathol..

[52] Tomita M., Matsuzaki Y., Edagawa M., Shimizu T., Hara M., Sekiya R., Onitsuka T. (2001). Association of mast cells with tumor angiogenesis in esophageal squamous cell carcinoma. Dis. Esophagus.

[53] Ribatti D., Guidolin D., Marzullo A., Nico B., Annese T., Benagiano V., Crivellato E. (2010). Mast cells and angiogenesis in gastric carcinoma. Int. J. Exp. Pathol..

[54] Ammendola M., Sacco R., Donato G., Zuccalà V., Russo E., Luposella M., Vescio G., Rizzuto A., Patruno R., de Sarro G. (2013). Mast cell positivity to tryptase correlates with metastatic lymph nodes in gastrointestinal cancer patients treated surgically. Oncology.

[55] Acikalin M.F., Oner U., Topçu I., Yaşar B., Kiper H., Colak E. (2005). Tumour angiogenesis and mast cell density in the prognostic assessment of colorectal carcinomas. Dig. Liver Dis..

[56] Gulubova M., Vlaykova T. (2009). Prognostic significance of mast cell number and microvascular density for the survival of patients with primary colorectal cancer. J. Gastroenterol. Hepatol..

[57] Peng S.H., Deng H., Yang J.F., Xie P.P., Li C., Li H., Feng D.Y. (2005). Significance and relationship between infiltrating inflammatory cell and tumor angiogenesis in hepatocellular carcinoma tissues. World J. Gastroenterol..

[58] Esposito I., Menicagli M., Funel N., Bergmann F., Boggi U., Mosca F., Bevilacqua G., Campani D. (2004). Inflammatory cells contribute to the generation of an angiogenic phenotype in pancreatic ductal adenocarcinoma. J. Clin. Pathol..

[59] Ibaraki T., Muramatsu M., Takai S., Jin D., Maruyama H., Orino T., Katsumata T., Miyazaki M. (2005). The relationship of tryptase- and chymase- positive mast cells to angiogenesis in stage I non-small cell lung cancer. Eur. J. Cardiothorac. Surg..

[60] Carlini M.J., Dalurzo M.C., Lastiri J.M., Smith D.E., Vasallo B.C., Puricelli L.I., Lauría de Cidre L.S. (2010). Mast cell phenotypes and microvessels in non-small cell lung cancer and its prognostic significance. Hum. Pathol..

[61] Ribatti D., Ennas M.G., Vacca A., Ferreli F., Nico B., Orru S., Sirigu P. (2003). Tumor vascularity and tryptase-positive mast cells correlate with a poor prognosis in melanoma. Eur. J. Clin. Investig..

[62] Benítez-Bribiesca L., Wong A., Utrera D., Castellanos E. (2001). The role of mast cell tryptase in neoangiogenesis of premalignant and malignant lesions of the uterine cervix. J. Histochem. Cytochem..

[63] Ranieri G., Patruno R., Lionetti A., di Summa A., Mattioli E., Bufo P., Pellecchia A., Ribatti D., Zizzo N. (2005). Endothelial area and microvascular density in a canine non-Hodgkin’s lymphoma: An interspecies model of tumor angiogenesis. Leuk. Lymphoma.

[64] Nico B., Mangieri D., Crivellato E., Vacca A., Ribatti D. (2008). Mast cells contribute to vasculogenic mimicry in multiple myeloma. Stem Cells Dev..

[65] Ribatti D., Finato N., Crivellato E., Marzullo A., Mangieri D., Nico B., Vacca A., Beltrami C.A. (2005). Neovascularization and mast cells with tryptase activity increase simultaneously with pathologic progression in human endometrial cancer. Am. J. Obstet. Gynecol..

[66] Claman H.N. (1989). On scleroderma. Mast cells, endothelial cells, and fibroblasts. JAMA.

[67] Ehara T., Shigematsu H. (2003). Mast cells in the kidney. Nephrology.

[68] Staemmler M. (1958). Theodor Fahr’s contribution to modern renal pathology. Medizinische.

[69] Lascano E.F. (1958). Mast cells in human tumors. Cancer.

[70] Beil W.J., Füreder W., Wiener H., Grossschmidt K., Maier U., Schedle A., Bankl H.C., Lechner K., Valent P. (1998). Phenotypic and functional characterization of mast cells derived from renal tumor tissues. Exp. Hematol..

[71] Tuna B., Yorukoglu K., Unlu M., Mungan M.U., Kirkali Z. (2006). Association of mast cells with microvessel density in renal cell carcinomas. Eur. Urol..

[72] Mohseni M.G., Mohammadi A., Heshmat A.S., Kosari F., Meysamie A.P. (2010). The lack of correlation between mast cells and microvessel density with pathologic feature of renal cell carcinoma. Int. Urol. Nephrol..

[73] Yoshino S., Kato M., Okada K. (1998). Evaluation of the prognostic significance of microvessel count and tumor size in renal cell carcinoma. Int. J. Urol..

[74] Nativ O., Sabo E., Reiss A., Wald M., Madjar S., Moskovitz B. (1998). Clinical significance of tumor angiogenesis in patients with localized renal cell carcinoma. Urology.

[75] MacLennan G.T., Bostwick D.G. (1995). Microvessel density in renal cell carcinoma: Lack of prognostic significance. Urology.

[76] Kirkali Z., Yorukoglu K., Ozkara E., Kazimoglu H., Mungan U. (2001). Proliferative activity, angiogenesis and nuclear morphometry in renal cell carcinoma. Int. J. Urol..

[77] Patruno R., Marech I., Zizzo N., Ammendola M., Nardulli P., Gadaleta C., Introna M., Capriuolo G., Rubini R.A., Ribatti D. (2014). c-Kit expression, angiogenesis, and grading in canine mast cell tumour: A unique model to study c-Kit driven human malignancies. Biomed. Res. Int..

[78] Vuky J., Isacson C., Fotoohi M., dela Cruz J., Otero H., Picozzi V., Malpass T., Aboulafia D., Jacobs A. (2006). Phase II trial of imatinib (Gleevec) in patients with metastatic renal cell carcinoma. Investig. New Drugs.

[79] Calvani N., Morelli F., Chiuri V., Gnoni A., Scavelli C., Fedele P., Orlando L., Maiello E., Lorusso V., Cinieri S. (2013). Prolonged exposure to tyrosine kinase inhibitors or early use of everolimus in metastatic renal cell carcinoma: Are the two options alike?. Med. Oncol..

